# Effects of Irrigation Practices and N Addition Rates on Wheat Nutrient Accumulation and Utilization in Dryland

**DOI:** 10.3390/plants15020264

**Published:** 2026-01-15

**Authors:** Cuiping Zhao, Kaiming Ren, Yuhao Sun, Qinglei Xie, Shuai Zhang, Mengqi Yang, Shanwei Wu, Ming Huang, Jinzhi Wu, Youjun Li

**Affiliations:** College of Agriculture, Henan University of Science and Technology, Luoyang 471023, China; zhaocuiping883193@163.com (C.Z.); 230410170051@stu.haust.edu.cn (K.R.); sunyuhao202410@163.com (Y.S.); xieqinglei101@163.com (Q.X.); zhangshuai089419@163.com (S.Z.); 18537265941@163.com (M.Y.); wsw1223@163.com (S.W.); huangming_2003@126.com (M.H.)

**Keywords:** dryland, wheat, one-off irrigation, N addition rate, yield, nutrient use efficiency

## Abstract

Irrigation practices and nitrogen (N) addition play pivotal roles in wheat production, and their rational coordination can significantly enhance N, phosphorus (P), and potassium (K) use efficiency and yield of wheat. However, the comprehensive effects of irrigation practices and N addition rates on N, P, and K accumulation and utilization and yield of wheat in dryland remain unclear. A field experiment with two irrigation practices (W0, zero-irrigation and W1, one-off irrigation), and four N addition rates (0, 120, 180, and 240 kg N ha^−1^, represented by N0, N120, N180, and N240, respectively) was conducted in 2021–2022 and 2023–2024. Compared to W0N0, W1N180 significantly increased wheat grain yield, spike number, and grains per spike by 46.4%, 35.9%, and 18.9%, respectively. Wheat yield and N, P, and K accumulation reached the maximum value at N180 or N240. One-off irrigation significantly improved the uptake efficiency and fertilizer partial factor productivity for N, P, and K, whereas increased N addition enhanced these parameters specifically for P and K. However, N180 treatment increased N uptake efficiency, N fertilizer partial factor productivity, P internal efficiency, and K internal efficiency by 22.2%, 31.1%, 9.4%, and 5.9%, respectively, compared to N240 under one-off irrigation. In addition, W1N180 significantly increased above-ground N, P, and K accumulation by 45.8%, 52.8%, and 51.8%, as well as pre-anthesis N and P translocation by 48.5% and 47.0%, respectively, compared to W0N120. Consequently, the W1N180 strategy not only improved wheat yield but also optimized N, P, and K accumulation, pre-anthesis N and P translocation, and nutrient use efficiency. Therefore, one-off irrigation combined with N180 can be recommended for enhancing wheat yield and nutrient use efficiency in dryland.

## 1. Introduction

Wheat (*Triticum aestivum* L.) is a critical source of carbohydrates, proteins, fats, vitamins, and minerals [1]. It provides approximately 20% of global dietary protein and carbohydrates intake for humankind, playing a pivotal role in safeguarding food security for humans [2]. In China, wheat is primarily distributed across northern China, one third of which is planted in dryland agricultural regions [3]. In these regions, wheat production persistently faces challenging natural conditions, including low and erratic precipitation, a lack of supplemental irrigation, and poor soil fertility [4]. Moreover, unsustainable farming practices, such as excessive N application, further constrain wheat productivity [5,6]. These issues led to a low and unstable yield, and poor resource efficiency in dryland wheat. In addition, N, P, and K are the three primary essential nutrients to ensure wheat growth and development, and their accumulation and utilization characteristics are closely related to growth rates and final grain yield [7]. Therefore, it is of great significance in seeking effective measures to improve N, P, and K accumulation and utilization characteristics in wheat for enhancing yields and ensuring food security.

Many studies have demonstrated that proper irrigation [8,9,10] and N addition [11,12,13] elevated grain yield through the optimizing of plant N, P, and K accumulation, translocation, allocation, and use efficiency of these nutrients. A study conducted in the semi-arid region of Egypt demonstrated that, under drip irrigation conditions, the N240 addition rate significantly increased the accumulation of N, P, and K by 66.3%, 48.6%, and 43.5%, respectively, compared to N180 [14,15]. Research in the Indian Plains also showed that a higher fertilizer rate markedly increased wheat N, P, and K uptake [10,16]. In addition, studies demonstrated that proper N addition rates optimized the partial factor productivity, internal efficiency, and agronomic efficiency of N, while simultaneously enhancing wheat yield [13,17]. Another study showed that, under sufficient water and fertilizer conditions, the stem, leaf, and rachis + glume of wheat tend to obtain a higher proportion of N and P, resulting in a decrease in the N and P harvest index [18]. In essence, irrigation practices and N addition rates interactively influenced the characteristics of N, P, and K accumulation and utilization in crops [19]. However, previous studies were mainly focused on the mono-cropping system in dryland regions or the wheat–maize double-cropping system in irrigation regions.

Water resources and irrigation infrastructure are scarce in dryland wheat production systems, leading to low and unstable yield and nutrient use efficiency. In recent years, the accelerated development of High-Standard Farmland infrastructure in China and worldwide has enabled many dryland fields to realize an irrigation opportunity. However, most dryland fields can only be irrigated one time during the whole wheat growth stage, a practice defined as “one-off irrigation” in this and our previous studies [3,20]. Many studies have proved that the one-off irrigation can increase wheat yield and nutrient utilization. For instance, a field experiment in Beijing, China, showed that applying one-off irrigation at the jointing stage increased wheat yield by 18.6% compared to zero-irrigation [21]. Research in dryland wheat and maize intercropping systems also confirmed that one-off irrigation optimized wheat yield components, thereby enhancing wheat yield [22,23]. Furthermore, a field experiment conducted in Creston, Massachusetts, USA revealed that one-off irrigation during the grain-filling stage significantly increased wheat yield [24]. However, compared to two, three, and four irrigations with same amount at each time, one-off irrigation decreased grain yield by 7.5%, 10.2%, and 11.6%, N accumulation by 17.1%, 32.2%, and 41.3%, P accumulation by 22.3%, 40.1%, and 50.8%, and K accumulation by 13.7%, 26.1%, and 33.3%, respectively, under the same N application rate [25]. Interestingly, our previous study showed one-off irrigation increased grain yield, N uptake efficiency, N agronomy efficiency, and N apparent use efficiency by 56.0%, 44.2%, 48.8%, and 58.5% compared to zero-irrigation [26]. Despite these findings, scant literature exists regarding the interactive effects of one-off irrigation and different N addition rates on N, P, and K accumulation and utilization dynamics in dryland wheat.

Therefore, we carried out a field experiment in a typical dryland wheat production region in China, which included two irrigation practices (zero-irrigation and one-off irrigation) and four N addition rates (0, 120, 180, and 240 kg N ha^−1^) in a typical dryland wheat production region in China. This study aimed to achieve the following: (1) quantify the effects of one-off irrigation paired with different N addition rates on wheat yield, N, P, and K accumulation, translocation, and allocation, and N, P, and K efficiencies; and (2) identify the optimal N addition rates for achieving high-yield and resource-efficient wheat production under one-off irrigation conditions. The findings would provide a scientific basis for formulating N management strategies to enhance wheat productivity in drylands where one-off irrigation is ensured.

## 2. Results

### 2.1. Yield and Yield Components

**Figure 1:**
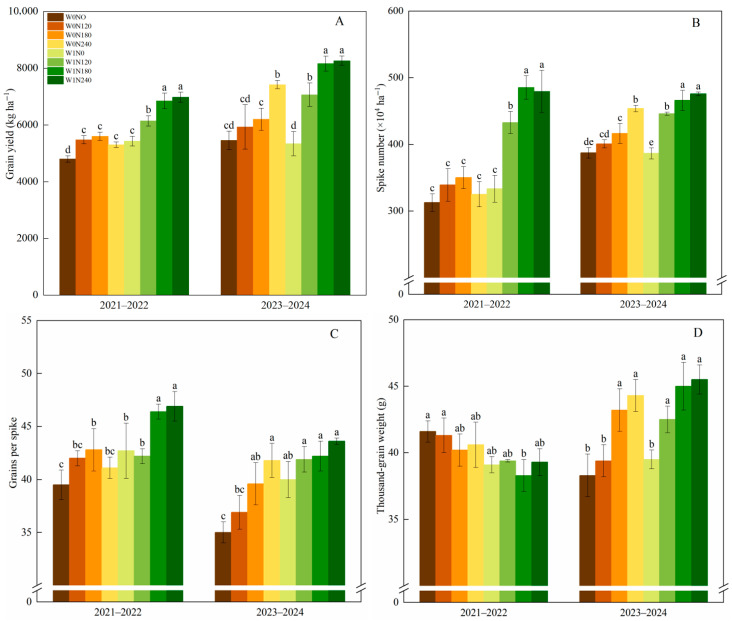
Effect of irrigation practices and N addition rates on grain yield (**A**), spike number (**B**), grains per spike (**C**), and thousand-grain weight (**D**) of wheat in dryland wheat. Note: W0, zero-irrigation; W1, one-off irrigation; N0, N120, N180, and N240 refer to N addition rates at 0, 120, 180, and 240 kg N ha^−1^, respectively. The error bar indicates standard deviation. Different lowercase letters within the same year indicate significant differences by Duncan’s test at *p* < 0.05.

Both the irrigation practices and N addition rates significantly affected wheat grain yield and its components in drylands (Figure 1). Compared to W0, W1 significantly increased grain yield by 19.9% and 15.3% in 2021–2022 and 2023–2024, respectively. Spike number and grains per spike followed similar trends to grain yield. The effect of N addition rates on grain yield varied depending on experimental years and irrigation practices. Under W0, grain yield peaked at N180 in 2021–2022, surpassing N0 and N240 by 16.7% and 5.7%, respectively. In contrast, in 2023–2024, N240 achieved the highest yield, exceeding N0, N120, and N180 by 36.0%, 25.2%, and 19.7%, respectively. Under W1, spike number, grains per spike (except for 2021–2022), and thousand-grain weight firstly increased and then stabilized. N240 had no significant difference from N180, but they were showing a significant superiority over N0 and N120. W1N180 resulted in increasing yield, spike number, and grains per spike by 46.4%, 35.9%, and 18.9% compared to W0N0.

### 2.2. Above-Ground N, P, and K Accumulation

Both irrigation practices and N addition rates significantly affected the accumulation of N, P, and K in wheat (*p* < 0.01). Moreover, their interaction significantly affected N accumulation at the jointing, anthesis, and maturity stages, and P and K accumulation at the maturity stage (Table 1). Specifically, under N240, W1 significantly increased N, P, and K accumulation by 27.5%, 48.8%, and 34.2%, respectively, compared to W0 over the two years. Under W1, the N, P, and K accumulation under N240 significantly increased at the maturity stage compared to other N treatments. Most notably, W1N180 significantly increased N, P, and K accumulation at the maturity stage by 66.4%, 116%, and 72.2% compared to W0N0.

### 2.3. N and P Accumulation and Translocation

**Figure 2:**
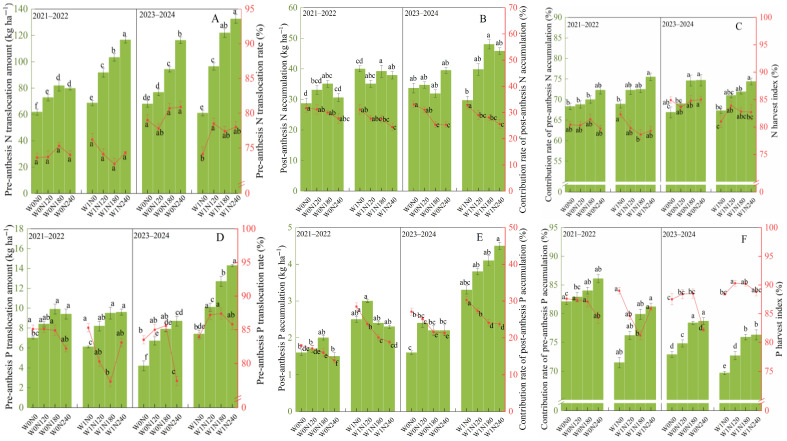
Effect of irrigation practices and N addition rates on N and P accumulation and translocation of dryland wheat. Note: W0, zero-irrigation; W1, one-off irrigation; N0, N120, N180, and N240 refer to N addition rates at 0, 120, 180, and 240 kg N ha^−1^, respectively. The error bar indicates standard deviation. Different lowercase letters within the same year indicate significant differences at *p* < 0.05. The capital letters correspond to the following: (**A**,**D**) indicate the pre-anthesis N and P translocation amount and their translocation rates, respectively; (**B**,**E**) indicate the post-anthesis N and P accumulation and their contribution rate, to grains, respectively; (**C**,**F**) indicate the contribution rate of pre-anthesis N and P accumulation and N and P harvest index, respectively.

As shown in Figure 2, across different N addition rates, W1 could significantly enhance the pre-anthesis N translocation amount compared to W0 in 2021–2022 and the pre-anthesis P translocation amount under N240 in two years. Under W1, N180 and N240 improved the pre-anthesis N translocation amount, and the post-anthesis N accumulation amount by 19.7–32.4%, and 11.7–16.4%, while they reduced contribution rates of post-anthesis N accumulation by 8.3–14.7% and N harvest index by 5.1–9.8% compared to N120. Likewise, under W1, both pre-anthesis P translocation amount and contribution rates of pre-anthesis P accumulation to grain increased with the increase in N addition rates, peaking at N240; however, N240 reduced the contribution rates of post-anthesis P accumulation to grain by 14.8–22.5% and P harvest index by 7.3–12.1% compared to N120 (Figure 2D,F). Considering the interaction effects, W1N180 significantly increased pre-anthesis N and P translocation and the contribution rates of pre-anthesis N accumulation to grain, and post-anthesis N and P accumulation compared to W0N0.

### 2.4. N, P, and K Accumulation and Allocation in Different Organ at Maturity

**Figure 3:**
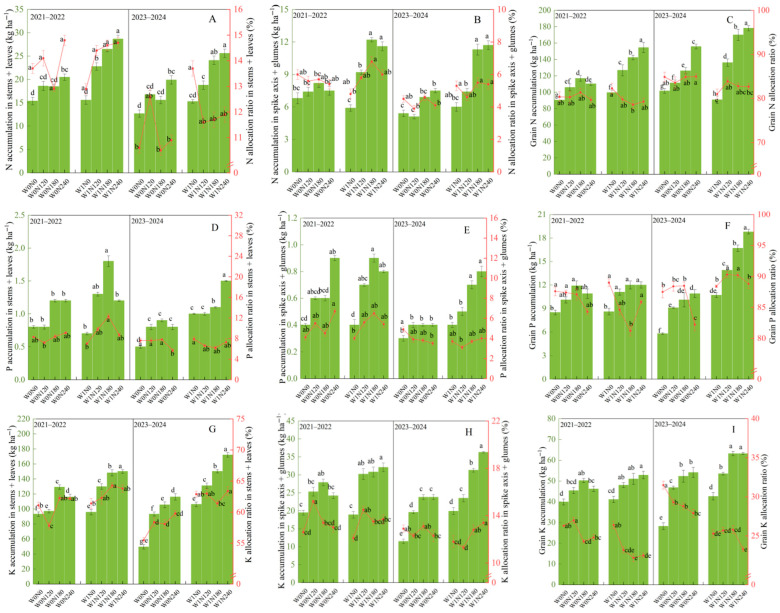
Effect of irrigation practices and N addition rates on N, P, and K accumulation and allocation in different organs in dryland wheat. Note: W0, zero-irrigation; W1, one-off irrigation; N0, N120, N180, and N240 refer to N addition rates at 0, 120, 180, and 240 kg N ha^−1^, respectively. The error bar indicates standard deviation. Different lowercase letters within the same year indicate significant differences by Duncan’s test at *p* < 0.05. The capital letters correspond to the following: (**A**,**D**,**G**) indicate the N, P, and K accumulation and their allocation ratios in stems + leaves; (**B**,**E**,**H**) indicate the N, P, and K accumulation and their allocation ratios in spike axis + glumes: (**C**,**F**,**I**) indicate the N, P, and K accumulation and their allocation ratios in grains.

Irrigation practices and N addition rates significantly influenced N accumulation and allocation ratios in different organs of wheat at the maturity stage (Figure 3). Compared to W0, W1 significantly enhanced grain N, P, and K accumulation in both of the two years. Under the same N addition rate, W1 increased the N and K allocation ratio in stems + leaves and spike axis + glumes, but reduced the N allocation ratio in grains, and the trends over two years were consistent (Figure 3A–C,G,H). In addition, W1 increased the grain P allocation ratio in 2023–2024, compared to W0 (Figure 3F). The W1N180 treatment did not affect N, P, and K accumulation at the maturity stage except for grain N and P accumulation and K accumulation in stems + leaves and spike axis + glumes, but it significantly enhanced N, P, and K accumulation across all measured organs compared to other treatments. Specifically, compared to W0N0, it increased N accumulation by 80.1% in stems + leaves, 92.6% in spike axis + glumes, and 62.3% in grains. Corresponding increases for P were 123.1%, 128.6%, and 100.7%, and for K, they were 108.9%, 100.3%, and 67.7%. However, the W1N180 treatment significantly decreased the grain N, P, and K allocation ratio compared to W0N0.

### 2.5. N, P, and K Use Efficiency in Wheat

As shown in Table 2, irrigation practices and N addition rates significantly affected N, P, and K efficiency, except that irrigation practices had no impact on N agronomy efficiency (NAE) or N internal efficiency (NIE). The interaction of irrigation practices and N addition rates significantly affected NAE, N (P and K) uptake efficiency (NUptE, PUptE, and KUptE) and N (P and K) fertilizer partial factor productivity (PFPN, PFPP, and PFPK). With the increase in N addition rates, NAE, NIE, NUptE, and PFPN gradually decreased, while the P and K use efficiency varied depending on irrigation practices and index. Averaged across the two years, W1 increased NUptE by 18.2–37.5% and PFPN by 17.5–27.1% compared to the W0 under the same N addition rate, while no significant differences were observed for NAE and NIE. Compared to W0, W1 consistently increased P uptake efficiency (PUptE), K uptake efficiency (KUptE), and K fertilizer partial factor productivity (PFPK). In contrast, it decreased K internal efficiency (KIE) in wheat over the two years. However, the NIE increased in 2021–2022 but decreased in 2023–2024. Finally, compared to the W1N240, W1N180 significantly increased NUptE, PFPN, PIE, and KIE by 22.2%, 31.1%, 9.4%, and 5.9%, respectively. These results indicated that one-off irrigation combined with N180 can increase N, P, and K use efficiency in dryland wheat.

### 2.6. Relationship Between Yield and N, P, and K Accumulation and Utilization in Dryland Wheat

**Figure 4:**
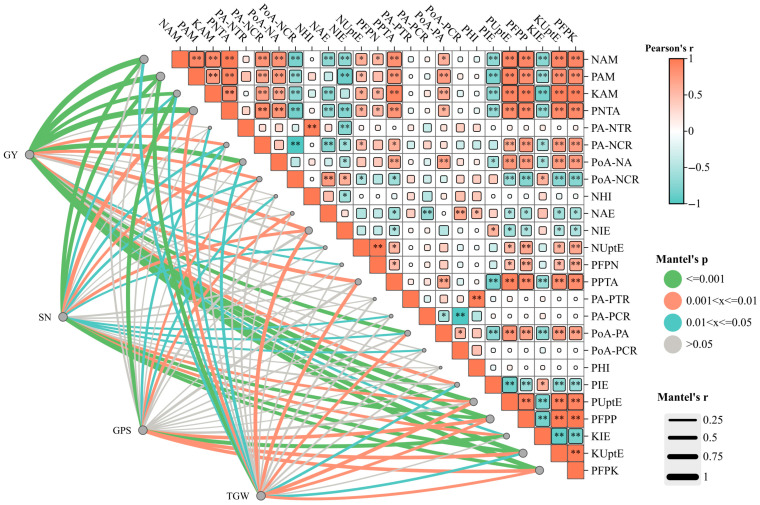
Correlation between yield, yield component, N, P, and K accumulation, translocation, allocation, and utilization of wheat in dryland wheat–maize double-cropping system. The Pearson’s correlation coefficients between main indices of wheat growth are shown by color gradients. The yield, spike number, grains per spike, and thousand-grain weight were related to main indices of wheat N, P, and K accumulation, translocation, and utilization efficiency by Mantel’s tests. Edge width corresponds to Mantel’s r statistic for the corresponding distance correlations, and edge color denotes the statistical significance. Note: GY: grain yield; SN: spike number; GPS: grain per spike; TGW: thousand-grain weight; NAM (PAM, KAM): N (P and K) accumulation at maturity; PN(P)TA: pre-anthesis N (P) translocation amount; PA-N(P)TR: pre-anthesis N (P) translocation rate; PA-N(P)CR: contribution rates of pre-anthesis N (P) translocation to grain N; PoA-N(P)A: post-anthesis N (P) accumulation; PoA-N(P)CR: contribution rates of post-anthesis N (P) accumulation to grain N; N(P)HI: N (P) harvest index; NAE: N agronomy efficiency; N (P and K)IE: N (P and K) internal efficiency; N (P and K)UptE: N (P and K) uptake efficiency; PFPN (P and K): N (P and K) fertilizer partial factor productivity. * and ** indicate significant effect at the level of *p* < 0.05 and *p* < 0.01, respectively.

As shown in Figure 4, wheat yield showed highly significant positive correlations (*p* < 0.01) with N, P, and K accumulation, as well as with multiple nutrient efficiency indices. These indices included the pre-anthesis N and P translocation amount, contribution rate of pre-anthesis N translocation to grain N, post-anthesis N and P accumulation, pre-anthesis P translocation, and P and K uptake efficiency. Furthermore, yield was positively correlated with N, P, and K fertilizer partial factor productivity. In addition, yield components such as spike number and thousand-grain weight were also positively associated with key nutrient metrics. These metrics included above-ground N, P, and K accumulation at the maturity stage, and pre-anthesis N translocation amount.

## 3. Discussion

### 3.1. Effects of Irrigation Practices and N Addition Rates on Yield in Dryland Wheat

Irrigation practices and N addition rates are two critical factors for optimizing wheat yield in dryland regions [23]. In our study, compared to W0N0, W1N180 significantly increased grain yield, spike number, and grains per spike by 46.4%, 35.9%, and 18.9%, respectively. This result demonstrated that one-off irrigation after the regreening stage helped to achieve high yield in wheat. The primary mechanisms may be ascribed to the improved topsoil water after regreening induced by the one-off irrigation, which promoted tiller survival and spikelet differentiation, thereby ensuring optimal population density [27]. A meta-analysis also showed that maintaining a proper soil water content from the regreening stage to the flowering stage could enhance population density and coordinated yield components, finally ensuring stable and high yields of wheat [28]. In the North China Plain, one-off irrigation at the jointing stage based on the water content of the 0–40 cm soil layer significantly increased grain yield by 48.2%, compared to that based on the water content of the 0–20 cm soil layer [29]. These studies indicated that the soil testing one-off irrigation based on the 0–40 cm soil layer is suitable for enhancing wheat yield in dryland. In contrast, under zero-irrigation, the limited rainfall during the wheat growing fails to meet crop water requirements, leading to massive tiller abortion and yield reduction [30]. A field study also reported that, in Lebanon, one-off irrigation at booting–flowering and grain-filling stages significantly increased thousand-grain weight and grain yield by 9.6% and 15.9%, compared to zero-irrigation [31].

**Figure 5:**
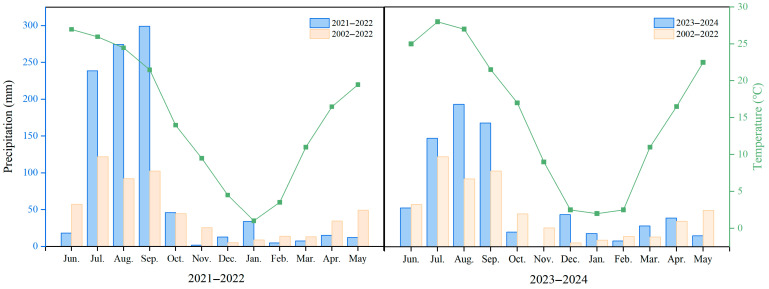
Monthly average temperature and precipitation at the experimental Site (2021–2022 and 2023–2024) and the average precipitation from 2002 to 2022.

Our results also showed that, under the zero-irrigation, the spike number, grains per spike, and grain yield generally followed a parabolic trend with the increase in N addition rate in 2021–2022. A potential explanation is that, under low N application rates, N fertilizer can significantly promote wheat growth, development, and photosynthetic capacity, thereby increasing grain yield [15]. However, when N input is excessively high, wheat plants may exhibit excessive vegetative growth and canopy overcrowding, which can impede light penetration and air circulation within the population [32]. During the 2023–2024 growing season, all of the grain yield, spike number, grain number per spike, and thousand-grain weight treatment increased with an increasing N addition rate. The reason may be that the experimental site in this study is located in a dryland agricultural system where water availability is the primary constraint on further increasing wheat yield. Figure 5 illustrates that the total precipitation during the 2023–2024 wheat growing season, particularly during the critical period from jointing to grain-filling, was higher than the 2021–2022 season and the average of the past two decades. Specifically, adequate precipitation in March to April improves water supply and increases the N availability, and finally ensures effective tillering and floret differentiation [33,34]. The most important thing is that the more sufficient and reasonably distributed precipitation in April to May during the grain filling period greatly increases wheat “source” production, finally alleviating the competition of “sink” and “source” and making grains plump [35,36]. Consequently, with the increase in N addition rates, the spike number, grains per spike, and thousand-grain weight increased simultaneously.

### 3.2. Effects of Irrigation Practices and N Addition Rates on N, P, and K Accumulation, Translocation, and Allocation in Dryland Wheat

Previous studies have demonstrated that one-off supplemental irrigation at the jointing stage significantly increased above-ground N accumulation at anthesis and enhanced pre-anthesis N reallocation to grains compared to zero-irrigation [37]. Our findings showed that, compared to zero-irrigation, one-off irrigation significantly increased plant N, P, and K accumulation at the jointing, anthesis, and maturity stages over the two years. Among these, N accumulation increased by 6.9%, 23.8%, and 21.4%, respectively; P accumulation increased by 46.2%, 24.7%, and 40.6%, respectively; and K accumulation increased by 9.1%, 35.9%, and 40.9%, respectively. This is primarily attributed to the irrigation-induced water improvement promoting nutrient dissolution and canopy transpiration, thereby enhancing nutrient uptake [38,39]. Moreover, irrigation optimized the activity of key enzymes involved in nutrient absorption, consequently increasing the N, P, and K accumulation across different growth stages of wheat [40].

The synergistic effects of irrigation practices and N addition rates facilitated N absorption and translocation capacity during early growth stages, while driving N remobilization and grain-directed allocation during later growth stages [41]. A study conducted in the North China Plain showed that both irrigation practices and N addition rates of 120 kg ha^−1^ increased N accumulation in vegetative organs at the anthesis stage and pre-anthesis N translocation to grains [42]. Our findings suggested that one-off irrigation combined with 180 kg N ha^−1^ addition rates enhances pre-anthesis N and P translocation and grain N, P, and K accumulation at maturity. The underlying mechanisms may include the following: (1) Adequate water availability improves photosynthetic efficiency, thereby promoting nutrient translocation to grains. (2) Enhanced N addition rates elevate the activities of key enzymes such as nitrates reductase and glutamine synthetase in wheat leaves, accelerating N assimilation processes. (3) Sufficient N not only enhances plant N synthesis capacity but also stimulates P uptake through the “N-P synergistic absorption” mechanism [43,44,45,46].

### 3.3. Effects of Irrigation Practices and N Addition Rates on N, P, and K Use Efficiency in Dryland Wheat

In dryland wheat production systems, the synergistic management of “regulating nutrients via water” and “enhancing water use via nutrients” was used to overcome the limitations of both water and nutrient stress [26,47]. This synergistic management facilitates the transformation of N, P, and K resources from soil immobilization to efficient crop use, which is of great significance for enhancing the nutrient use efficiency of wheat in drylands. The results in the present study indicated that NAE, NIE, NUptE, PFPN, and PIE declined with an increase in N addition rates. However, W1 significantly increased NUptE, PFPN, PUptE, PFPP, KUptE, and PFPK in wheat over the two years, compared to W0. The possible reason is that the implementation of one-off irrigation directly enhanced soil water content [48]. This increased soil water content promoted wheat root development and the availability of soil N, P, and K elements, thereby establishing a foundation for improved nutrient absorption and utilization by the wheat plants [25,49]. Finally, compared to the W1N240, W1N180 significantly increased NUptE, PFPN, PIE, and KIE in dryland wheat. The reason may be that the over-addition of N disrupted the carbon–nitrogen metabolic balance during grain-filling stage of wheat [50]. The enhanced N metabolism consumes plant nutrients, weakening the translocation of carbohydrates and nutrients to the grains [51,52]. In addition, under the same irrigation practice, optimal N addition enhanced root vitality, thereby promoting nutrient uptake efficiency in wheat [53]. Previous studies have also demonstrated that proper N addition significantly improved the NIE and KIE under irrigation [54]. These results indicated that one-off irrigation combined with N180 can partly increase N, P, and K use efficiency in dryland wheat.

## 4. Materials and Methods

### 4.1. Experimental Site Description and Precipitation Patterns

The field experiment was conducted in 2021–2022 and 2023–2024, based on a filed positioning experiment which initiated in 2019 and was located in Yichuan County, Luoyang City, Henan Province, China, the junction of the Loess Plateau and the Huang-Huai-Hai Plain. The experimental site is in a typical dryland wheat production region in China, with a temperate continental monsoon climate, with an average annual precipitation of 663.4 mm, and a mean annual air temperature of 14.5 °C. The specific monthly precipitation and average temperature in the experimental years are shown in Figure 5. The soil falls under the category of heavy loam according to the FAO (1993) [55]. At the initiation of the experiment in 2019, the topsoil (0–20 cm layer) exhibited the following properties: pH of 8.0, organic matter content of 12.4 g kg^−1^, total N content of 1.1 g kg^−1^, available P content of 12.7 mg kg^−1^, bulk density of 1.40 g cm^−3^, maximum field capacity of 26.0%, proportion of <0.01 mm soil particles of 22.8%, and available K content of 177.1 mg kg^−1^.

### 4.2. Experimental Design and Field Management

The experiment was conducted using a two-factor split-plot design with irrigation practice as the main plot treatment, and N addition rates as the subplot treatment. Two irrigation practices were zero-irrigation (W0) and one-off irrigation (W1, irrigated to 85% of field capacity when the soil water content in the 0–40 cm soil layer was lower than a threshold—60% of field capacity after wheat regreening). In 2021–2022 and 2023–2024, the soil water content of the 0–40 cm soil layer before irrigation was 15.1% and 14.7%, and the irrigation amount was 37.8 mm and 39.9 mm, respectively. The secondary factor included four N rates, which consisted of 0 (N0), 120 (N120), 180 (N180), and 240 (N240) kg N ha^−1^. For W1, soil moisture in the 0–40 cm layer was measured every 3 days after regreening, with irrigation triggered immediately when moisture fell below the threshold. Actual irrigation volumes were measured using water meters, and the irrigation amount (mm) was calculated using the water balance method:

Irrigation amount (mm) = 10 × ρb × H × (θi − θj)

where ρb is the average soil bulk density of the wetting layer (g·cm^−3^); H is the planned wetting depth (cm); θi is the target soil moisture content (85% of field capacity), and θj is the actual soil moisture content before irrigation (%).

Each treatment had three replications with plot dimensions of 4 m × 8 m (32 m^2^). Chemical fertilizers included urea (46% N), calcium superphosphate (12% P_2_O_5_), and potassium sulfate (50% K_2_O). The P and K fertilizer with the rates of 90 kg P_2_O_5_ ha^−1^ and 60 kg K_2_O ha^−1^ was uniform for all treatments. For W0, all N, P, and K fertilizers were handily broadcast before sowing and incorporated into soil through rotary tillage (15 cm depth). For W1, 50% of N and all the P and K were applied as basal as W0, and the remaining 50% N fertilizer was applied as topdressing along with the one-off irrigation. The wheat variety “Luohan 22” was planted with a seeding rate of 187.5–225.0 kg ha^−1^. The wheat was sown on 25 October 2021, and harvested on 2 June 2022, in the 2021–2022 season, and on 17 October 2023 and 31 May 2024, in the 2023–2024 season, weeds, pests, and diseases were controlled with herbicides and pesticides according to local practices.

### 4.3. Measurements and Methods

#### 4.3.1. Yield and Yield Components

At the maturity stage, four random quadrats covering 1 m^2^ (1 m × 1 m) were randomly selected and harvested from each plot. Plants within quadrats in each plot were mixed, air-dried, threshed, and weighed. Then, a subsample of grains (50 ± 5 g) was oven-dried at 70 °C to a constant weight to determine grain moisture content. Grain yield (kg ha^−1^) was calculated by adjusting the measured yield to a standardized moisture content of 12.5%. Additionally, 50 randomly selected wheat plants in each plot were used to quantify grains per spike and thousand-grain weight.

#### 4.3.2. N, P, and K Accumulation, Translocation, and Allocation

At the jointing, anthesis, and maturity stages in 2021–2022 and 2023–2024, 50 wheat plants were randomly sampled from each plot. After cutting off the root, above-ground tissues were further processed. The sampled plants at the anthesis stage were separated into stem-sheath-leaves (hereafter referred to stem-leaves) and spikes, while those at the maturity stage were divided into stems-leaves, spike axis and glumes, and grains. All plant materials underwent initial dehydration at 105 °C for 30 min, and were then oven-dried at 75 °C to a constant weight for determining the dry matter weight. The oven-dried samples were pulverized using a ball miller (MM400, RETSCH, Haan, Germany) and then digested with H_2_SO_4_-H_2_O_2_ [56]. The N and P concentrations (g mL^−1^) were determined using an AutoAnalyzer 3 (AA3, Seal Company, Norderstedt, Germany), and K concentration (g mL^−1^) was measured using a flame spectrophotometer (Flame Photometer 410, Sherwood Company, Buckinghamshire, UK). The N, P, and K accumulation, translocation, and allocation were calculated [26,27,57,58,59]. The calculations were as follows:

N (P and K) accumulation (kg ha^−1^) = Dry weight × N (P and K) content (g kg^−1^)

Above-ground N (P and K) accumulation (kg ha^−1^) = N (P and K) accumulation in stems-leaf-sheaths + spike axis-glumes + N (P and K) accumulation in grains

Pre-anthesis N (P) translocation (kg ha^−1^) = Above-ground N (P) accumulation at anthesis − Above-ground N (P) accumulation of vegetative organs at maturity

Pre-anthesis N (P) translocation rate (%) = Pre-anthesis N (P) translocation ÷ Above-ground N (P) accumulation at anthesis × 100

Contribution rate of pre-anthesis N (P) translocation to grain N (P) (%) = Pre-anthesis N (P) translocation ÷ Grain N (P) accumulation at maturity × 100

Post-anthesis N (P) accumulation amount (kg ha^−1^) = Grain N (P) accumulation at maturity − Above-ground N (P) accumulation at anthesis

Contribution rate of post-anthesis N (P) accumulation to grain N (P) (%) = Post-anthesis N (P) ÷ Grain N (P) accumulation at maturity × 100

N (P and K) harvest index (%) = Grain N (P and K) accumulation at maturity (kg ha^−1^) ÷ Above-ground N (P and K) accumulation at maturity (kg ha^−1^) × 100

N (P and K) allocation ratio = N (P and K) accumulation in the organ ÷ N (P and K) accumulation in the total above − ground part

#### 4.3.3. Nutrient Use Efficiency

The nutrient use efficiency was calculated [59,60]. The calculations were as follows:

N (P and K) uptake efficiency (kg kg^−1^) = Above-ground N (P and K) accumulation at maturity (kg ha^−1^) ÷ Fertilizer N (P and K) addition rate (kg ha^−1^)

N (P and K) partial factor productivity (kg kg^−1^) = Grain yield under N (P and K) application treatment ÷ N (P and K) addition rate (kg ha^−1^)

N agronomy efficiency (%) = N accumulation in N applied treatment − N accumulation in N0 treatment)] ÷ N addition rate × 100

The N, P, K internal efficiency was calculated according to [61,62]. The calculations were as follows:

N (P, K) internal efficiency = Grain yield under N (P and K) application treatment ÷ N (P and K) accumulation in N (P and K) applied treatment

### 4.4. Statistical Analysis

Data were processed using Microsoft Excel 2019 to calculate means and standard deviations. Statistical analysis of inter-treatment differences was conducted with SPSS 23 (version 26, IBM Corp., Chicago, IL, USA) through one-way ANOVA followed by Duncan’s multiple range test (*p* < 0.05). All figures and tables were generated using Microsoft Excel 2019.

## 5. Conclusions

Our trial showed that, compared to zero-irrigation, one-off irrigation significantly improved the grain yield, as well as the accumulation of N, P, and K at both the anthesis and maturity stages. Additionally, it enhanced pre-anthesis N and P translocation, the contribution of post-anthesis N and P accumulation to the grain, and the NUptE, PUptE, KUptE, PFPN, and PFPK. In addition, one-off irrigation decreased the pre-anthesis N and P accumulation contribution rate to grain, N and P harvest index, and KIE. Adding 180 or 240 kg N ha^−1^ under one-off irrigation both resulted in a high grain yield and N, P, and K accumulation in wheat, but no significant difference was observed between the two treatments. Compared to W1N240, W1N180 significantly decreased N, P, and K accumulation but increased NUptE, PFPN, PIE, and KIE. Therefore, one-off irrigation with 180 kg N ha^−1^ was the optimal strategy for achieving high-yield and high-efficiency wheat production in drylands where one-off irrigation is assured in China and in similar conditions worldwide.

## Figures and Tables

**Table 1:** Effect of irrigation practices and N addition rates on above-ground N, P, and K accumulation of wheat in dryland wheat at jointing, anthesis, and maturity stages.

Year	Treatment	N Accumulation (kg ha^−1^)			P Accumulation (kg ha^−1^)			K Accumulation (kg ha^−1^)		
		Jointing	Anthesis	Maturity	Jointing	Anthesis	Maturity	Jointing	Anthesis	Maturity
2021–2022	W0N0	37.9 ± 6.3 e	83.9 ± 2.9 g	112.7 ± 5.1 g	4.8 ± 0.7 c	8.2 ± 0.5 cd	8.5 ± 0.5 d	89.9 ± 7.6 d	139.1 ± 10.1 d	138.3 ± 1.3 e
	W0N120	39.0 ± 4.3 de	98.9 ± 1.3 e	131.9 ± 3.6 e	5.1 ± 0.4 c	9.9 ± 2.3 bc	10.9 ± 1.1 cd	92.9 ± 15.7 cd	145.8 ± 11.6 d	151.7 ± 7.3 de
	W0N180	53.1 ± 4.3 b	108.6 ± 3.1 d	143.7 ± 4.7 d	7.4 ± 1.3 ab	11.7 ± 1.1 ab	11.9 ± 2.1 bc	135.6 ± 14.7 ab	194.8 ± 11.1 b	165.7 ± 5.7 d
	W0N240	53.7 ± 1.7 b	107.8 ± 0.9 d	138.4 ± 3.2 de	6.5 ± 1.9 bc	11.5 ± 2.0 ab	11.3 ± 1.6 bcd	116.0 ± 23.4 bc	185.7 ± 8.7 bc	145.9 ± 6.1 e
	W1N0	44.8 ± 2.6 cd	90.2 ± 2.6 f	121.2 ± 3.6 f	5.9 ± 1.5 bc	7.1 ± 0.3 d	9.6 ± 1.1 cd	102.9 ± 6.2 cd	162.3 ± 2.5 cd	164.0 ± 0.5 d
	W1N120	49.9 ± 1.2 bc	123.7 ± 7.1 c	158.8 ± 6.4 c	8.0 ± 0.9 ab	10.1 ± 0.8 abc	12.2 ± 1.5 bc	116.2 ± 7.6 bc	194.3 ± 19.4 b	197.1 ± 4.5 c
	W1N180	62.4 ± 2.1 a	142.1 ± 2.9 b	181.3 ± 3.2 b	8.7 ± 1.2 a	12.3 ± 0.6 a	13.8 ± 1.6 ab	138.6 ± 9.4 ab	237.0 ± 18.7 a	227.9 ± 14.6 b
	W1N240	61.3 ± 2.1 a	157.6 ± 3.1 a	194.7 ± 5.8 a	9.3 ± 1.0 a	11.6 ± 0.5 ab	15.4 ± 1.8 a	144.8 ± 15.3 a	247.9 ± 17.4 a	245.8 ± 11.0 a
2023–2024	W0N0	56.6 ± 2.3 f	86.0 ± 3.9 f	119.7 ± 3.0 g	3.7 ± 0.2 h	5.0 ± 0.4 f	6.6 ± 0.1 g	87.5 ± 3.9 e	91.8 ± 2.1 h	89.3 ± 3.4 h
	W0N120	85.3 ± 0.0 bc	98.7 ± 3.5 e	133.4 ± 4.3 f	5.2 ± 0.2 g	7.9 ± 0.4 e	10.3 ± 1.2 f	133.7 ± 6.0 b	164.5 ± 5.7 g	159.6 ± 6.7 g
	W0N180	88.9 ± 0.9 ab	116.7 ± 4.0 d	148.6 ± 1.8 e	5.9 ± 0.1 f	9.2 ± 0.4 d	11.4 ± 0.9 ef	138.4 ± 2.0 b	187.6 ± 2.9 e	181.7 ± 4.9 e
	W0N240	90.5 ± 3.8 a	143.8 ± 3.5 c	183.3 ± 3.8 c	6.2 ± 0.1 e	11.2 ± 0.1 c	13.4 ± 1.4 d	148.6 ± 2.9 a	205.7 ± 2.4 d	194.1 ± 2.8 d
	W1N0	67.9 ± 2.8 e	82.3 ± 2.5 f	112.1 ± 4.3 g	6.6 ± 0.1 d	8.8 ± 0.4 d	12.1 ± 1.1 de	99.7 ± 2.4 d	176.0 ± 5.5 f	168.8 ± 2.9 f
	W1N120	79.0 ± 1.1 d	122.6 ± 9.6 d	162.4 ± 6.6 d	7.8 ± 0.0 c	11.6 ± 0.4 c	15.4 ± 1.0 c	123.2 ± 3.1 c	218.4 ± 7.3 c	208.0 ± 6.4 c
	W1N180	83.6 ± 1.0 c	157.3 ± 7.4 b	205.4 ± 9.0 b	9.1 ± 0.1 b	14.5 ± 0.4b	18.6 ± 0.9 b	149.3 ± 2.7 a	261.1 ± 8.1 b	244.7 ± 3.9 b
	W1N240	91.1 ± 4.0 a	169.7 ± 4.6 a	215.5 ± 4.2 a	10.0 ± 0.0 a	16.7 ± 0.3 a	21.2 ± 0.9 a	153.4 ± 5.9 a	290.5 ± 3.3 a	271.5 ± 5.8 a
2-year average	W0N0	47.2 ± 2.3 e	85.0 ± 3.3 f	116.2 ± 3.8 f	4.2 ± 0.4 d	6.6 ± 0.2 d	7.5 ± 0.3 e	88.7 ± 5.8 e	115.5 ± 6.1 g	113.8 ± 1.6 f
	W0N120	62.1 ± 2.2 c	98.8 ± 1.4 e	132.6 ± 3.9 e	5.1 ± 0.3 d	8.9 ± 1.0 c	10.6 ± 1.1 d	113.3 ± 7.1 c	155.2 ± 7.9 f	155.7 ± 4.8 e
	W0N180	71.0 ± 2.6 b	112.7 ± 1.5 d	146.1 ± 2.8 d	6.7 ± 0.7 c	10.4 ± 0.6 b	11.7 ± 1.4 d	137.0 ± 8.1 b	191.2 ± 6.6 d	173.7 ± 3.5 d
	W0N240	72.1 ± 2.3 b	125.8 ± 2.2 c	160.8 ± 1.3 c	6.3 ± 1.0 c	11.3 ± 1.0 b	12.3 ± 1.4 cd	132.3 ± 11.0 b	195.7 ± 5.5 cd	170.0 ± 2.0 d
	W1N0	56.3 ± 1.7 d	86.3 ± 2.3 f	116.6 ± 3.7 f	6.2 ± 0.8 c	8.0 ± 0.3 c	10.9 ± 0.7 d	101.3 ± 2.7 d	169.1 ± 3.4 e	166.4 ± 1.2 d
	W1N120	64.4 ± 0.4 c	123.1 ± 5.4 c	160.6 ± 6.0 c	7.9 ± 0.5 b	10.8 ± 0.4 b	13.8 ± 0.5 c	119.7 ± 2.3 c	206.4 ± 6.7 c	202.5 ± 1.8 c
	W1N180	73.0 ± 1.1 b	149.7 ± 4.5 b	193.3 ± 3.9 b	8.9 ± 0.6 ab	13.4 ± 0.3 a	16.2 ± 0.7 b	144.0 ± 5.5 ab	249.1 ± 13.3 b	236.3 ± 9.0 b
	W1N240	76.2 ± 1.3 a	163.7 ± 1.5 a	205.1 ± 4.4 a	9.6 ± 0.5 a	14.2 ± 0.1 a	18.3 ± 1.2 a	149.0 ± 6.0 a	269.2 ± 10.0 a	258.7 ± 2.6 a
F-value	Y	1222.2 **	38.2 **	76.6 **	0.3 ns	1.5 ns	27.2 **	17.6 **	14.1 **	29.7 **
	W	26.0 **	372.9 **	460.4 **	111.4 **	76.4 **	134.5 **	13.7 **	398.5 **	1130.4 **
	N	139.4 **	390.7 **	437.1 **	27.1 **	86.4 **	51.3 **	57.7 **	190.7 **	317.4 **
	Y × W	24.7 **	7.0 *	2.9 ns	5.1 **	77.0 **	34.1 **	4.8 *	25.8 **	5.5 *
	Y × N	17.4 **	22.8 **	30.0 **	0.5 ns	7.4 **	4.5 **	4.6 **	12.4 **	43.1 **
	W × N	3.6 *	43.0 **	58.9 **	1.4 ns	2.1 ns	3.0 *	0.7 ns	2.9 ns	24.7 **
	N × W × Y	7.9 **	6.5 **	12.0 **	1.1 ns	0.6 ns	0.2 ns	2.8 ns	3.9 *	18.6 **

Note: W0, zero-irrigation; W1, one-off irrigation; N0, N120, N180, and N240 refer to N addition rates at 0, 120, 180, and 240 kg N ha^−1^, respectively. Data presented as mean *±* SD (*n* = 3). Different lowercase letters within the same column indicate significant differences at *p* < 0.05. Y, W, and N represent the variances of experimental year, irrigation method, and N addition rates, respectively. The symbols *, **, and ns indicate that the *p* values are <0.05, <0.01, and >0.05, respectively.

**Table 2:** Effect of irrigation practices and N addition rates on N uptake and utilization of wheat in dryland wheat–maize double-cropping system.

Year	Treatment	NAE	NIE	NUptE	PFPN	PIE	PUptE	PFPP	KIE	KUptE	PFPK
		(%)	(kg kg^−1^)	(kg kg^−1^)	(kg kg^−1^)	(kg kg^−1^)	(kg kg^−1^)	(kg kg^−1^)	(kg kg^−1^)	(kg kg^−1^)	(kg kg^−1^)
2021–2022	W0N0	42.6 ab	53.1 a	—	—	565.8 a	0.22 d	122.1 d	34.7 abc	2.78 e	96.4 d
	W0N120	41.5 bc	51.8 ab	1.10 b	45.6 b	504.1 b	0.28 bcd	139.4 c	36.2 ab	3.05 de	110.0 c
	W0N180	39.0 cd	48.0 bc	0.80 d	31.1 d	480.0 c	0.30 bc	142.5 c	33.8 bc	3.33 d	112.5 c
	W0N240	38.3 d	48.0 bc	0.60 e	22.1 f	475.8 c	0.29 bcd	134.9 c	36.4 a	2.93 e	106.4 c
	W1N0	44.8 a	54.5 a	—	—	568.7 a	0.25 cd	138.2 c	33.1 cd	3.29 d	109.0 c
	W1N120	38.7 cd	48.5 bc	1.30 a	51.2 a	506.4 b	0.31 bc	156.4 b	31.2 de	3.96 c	123.4 b
	W1N180	37.8 d	48.1 bc	1.00c	38.0 c	500.9 b	0.35 ab	174.3 a	30.1 ef	4.58 b	137.5 a
	W1N240	35.9 d	45.2 c	0.80 d	29.1 e	456.1 d	0.39 a	177.6 a	28.4 f	4.94 a	140.1 a
2023–2024	W0N0	44.2 ab	45.5 ab	—	—	526.5 a	0.17 f	88.3 de	38.9 a	1.79 h	69.7 de
	W0N120	43.2 abc	44.4 abc	1.10 b	48.0 b	552.0 a	0.26 e	143.5 cd	35.4 ab	3.21 g	113.3 cd
	W0N180	41.7 bcd	41.7 abc	0.80 d	34.4 c	538.6 a	0.29 de	155.9 bc	33.7 bc	3.65 e	123.0 bc
	W0N240	40.5 bcd	35.0 d	0.80 e	30.9 d	476.0 b	0.34 d	161.4 b	32.7 bcd	3.90 d	127.4 b
	W1N0	45.9 a	47.8 a	—	—	432.8 cd	0.31 d	132.4 c	30.9 cd	3.39 f	104.5 c
	W1N120	43.6 abc	43.6 abc	1.40 a	58.9 a	441.8 bc	0.39 c	172.3 b	32.6 bcd	4.18 c	135.9 b
	W1N180	39.8 cd	39.8 bcd	1.10 b	45.3 b	401.9 de	0.47 b	189.5 a	30.4 cd	4.91 b	149.5 a
	W1N240	38.4 d	38.4 cd	0.90 c	34.4 c	368.9 e	0.54 a	198.6 a	28.7 d	5.45 a	156.7 a
2-year average	W0N0	43.4 ab	49.3 ab	—	—	546.1 a	0.19 e	105.2 e	36.8 a	2.29 f	83.0 e
	W0N120	42.4 bc	48.1 bc	1.10 b	46.8 b	528.0 ab	0.27 d	141.4 cd	35.8 ab	3.13 e	111.6 cd
	W0N180	40.3 cd	44.8 d	0.80 d	32.8 d	509.3 bc	0.30 d	149.2 c	33.8 c	3.49 d	117.7 c
	W0N240	39.4 de	41.5 e	0.70 e	26.5 e	475.9 d	0.31 cd	148.1 c	34.5 bc	3.41 d	116.9 c
	W1N0	45.4 a	51.1 a	—	—	500.8 c	0.28 d	135.3 d	32.0 d	3.34 d	106.7 d
	W1N120	41.1 bcd	46.0 cd	1.30 a	55.0 a	474.1 d	0.35 c	164.3 b	31.9 d	4.07 c	129.7 b
	W1N180	38.8 de	43.9 de	1.10 b	41.7 c	451.4 e	0.41 b	181.9 a	30.3 de	4.75 b	143.5 a
	W1N240	37.1 e	41.7 e	0.90 c	31.8 d	412.5 f	0.47 a	188.1 a	28.6 e	5.19 a	148.4 a
F-value	Y	18.5 **	89.1 **	52.8 **	130 **	74.8 **	25.6 **	11.7 **	0.02	29.3 **	11.8 **
	W	2.1	0.1	428 **	215 **	143.0 **	127.3 **	230.8 **	73.9 **	1.12 **	230.8 **
	N	24.9 **	20.6 **	4061 **	3180 **	53.3 **	48.0 **	113.3 **	6.67 **	315.4 **	113.4 **
	Y × W	0.30 ns	1.30 ns	0.10 ns	3.60 ns	151.3 **	32.1 **	4.79 *	0.00 ns	5.53 *	4.79 **
	Y × N	0.50 ns	1.00 ns	14.2 **	15.9 **	14.4 **	4.34 *	20.7 **	1.13 ns	43.1 **	20.7 **
	W × N	3.00 *	1.10 ns	51.3 **	28.2 **	0.68 ns	3.00 *	2.91 *	1.05 ns	24.6 **	2.92 *

Note: W0, zero-irrigation; W1, one-off irrigation; N0, N120, N180, and N240 refer to 0, 120, 180, and 240 kg N ha^−1^, respectively. NAE: N agronomy efficiency; NIE: N internal efficiency; NUptE: N uptake efficiency; PFPN: N fertilizer partial factor productivity; PIE: P internal efficiency; PUptE: P uptake efficiency; PFPP: P fertilizer partial factor productivity; KIE: K internal efficiency; KUptE: K uptake efficiency; PFPK: K fertilizer partial factor productivity. Different lowercase letters within columns indicate significant differences by Duncan’s test at *p* < 0.05. Y, W, and N represent the variances of experimental year, irrigation method, and N addition rate, respectively. The symbols *, **, and ns indicate that the *p* values are <0.05, <0.01, and >0.05, respectively.

## Data Availability

The original contributions presented in this study are included in the article. Further inquiries can be directed to the corresponding author.
